# Ten interleukins and risk of prostate cancer

**DOI:** 10.3389/fonc.2023.1108633

**Published:** 2023-01-17

**Authors:** Bing-Hui Li, Si-Yu Yan, Li-Sha Luo, Xian-Tao Zeng, Yong-Bo Wang, Xing-Huan Wang

**Affiliations:** ^1^ Department of Urology, Zhongnan Hospital of Wuhan University, Wuhan, China; ^2^ Center for Evidence-Based and Translational Medicine, Zhongnan Hospital of Wuhan University, Wuhan, China

**Keywords:** interleukin, prostate cancer, Mendelian randomization, risk, causal inference

## Abstract

**Background:**

Interleukins (ILs) have been reported to be related to prostate cancer. The aims of this study were to estimate the levels for several key interleukins in prostate cancer and the causal effects between them.

**Methods:**

We conducted a bi-directional two-sample Mendelian randomization (MR) study to assess the causal associations between ILs and prostate cancer. Genetic instruments and summary-level data for 10 ILs were obtained from three genome-wide association meta-analyses. Prostate cancer related data were obtained from the PRACTICAL (79,148 cases and 61,106 controls), UK Biobank (7,691 cases and 169,762 controls) and FinnGen consortium (10,414 cases and 124,994 controls), respectively.

**Results:**

The odds ratio of prostate cancer was 0.92 (95% confidence interval (CI), 0.89, 0.96; *P=*1.58×10^-05^) and 1.12 (95% CI, 1.07, 1.17; *P=*6.61×10^-07^) for one standard deviation increase in genetically predicted IL-1ra and IL-6 levels, respectively. Genetically predicted levels of IL-1ß, IL-2a, IL-6ra, IL-8, IL-16, IL-17, IL-18, and IL-27 were not associated with the risk of prostate cancer. Reverse MR analysis did not find the associations between genetic liability to prostate cancer and higher levels of IL-1ra (β, -0.005; 95% CI, -0.010, 0.001; *P*=0.111) and IL-6 (β, 0.002; 95% CI, -0.011, 0.014; *P*=0.755).

**Conclusion:**

This MR study suggests that long-term IL-6 may increase the risk of prostate cancer and IL-1ra may reduce it.

## 1 Introduction

Prostate cancer is the second most common cancer in men and the fifth most prominent reason for cancer death worldwide (1). The global incident cases in 2019 increased by 169.11% for prostate cancer compared with 1990 according to the Global Burden of Disease 2019 database (2). The incidence of prostate cancer ranges from 6.3 to 83.4 per 100,000 men by region, with the highest rates in Northern and Western Europe, and the lowest rates in Asia and North Africa (3). For a disease as burdensome as prostate cancer, its causes and pathogenesis remain largely unknown. Therefore, it is necessary to search for more biomarkers to aid in the prevention, diagnosis and treatment of prostate cancer.

Studies have shown that the pro‐inflammatory cytokines may undertake important roles in promoting tumor cell proliferation and Interleukins (ILs) might be remarkable indicators in the proliferation and aggression of prostate cancer cells (4, 5). Previous studies have reported that the levels of IL-6 were higher in patients with prostate cancer (6–8). Moreover, IL-1ra, IL-8, IL-16, and several other interleukins were also researched in prostate cancer, but there were still lacked consistent opinions and a comprehensive evaluation for them in prostate cancer (9–12). Meanwhile, the causal associations between ILs and prostate cancer remain undetermined due to the non-negligible limitations of observational studies (such as reverse causality and residual confounding) and lack of high-level researches from randomized controlled trials (RCTs). Therefore, a pooled analysis including more ILs with a larger sample size is needed to assess the interactions between ILs and prostate cancer.

Mendelian randomization (MR) is a method to deal with observational bias. MR uses instrumental variables (IVs), where genetic variants are the instruments, alleles are randomly assigned during pregnancy, similar to the random assignment of treatment and control groups in RCTs (13, 14). Because genetic variants are not associated with confounders, differences in outcomes between variants carriers and noncarriers could be attributed to the differences in risk factors or disease susceptibility. Hence, compared with traditional observational studies that are susceptible to reverse causality or confounders, MR provides a robust estimation of the effects of modifiable exposures on the trait of interest. Therefore, we performed a wide-ranged MR study to explore the causal associations of 10 ILs with the risk of prostate cancer.

## 2 Methods

### 2.1 Study design and MR assumptions

**Figure 1:**
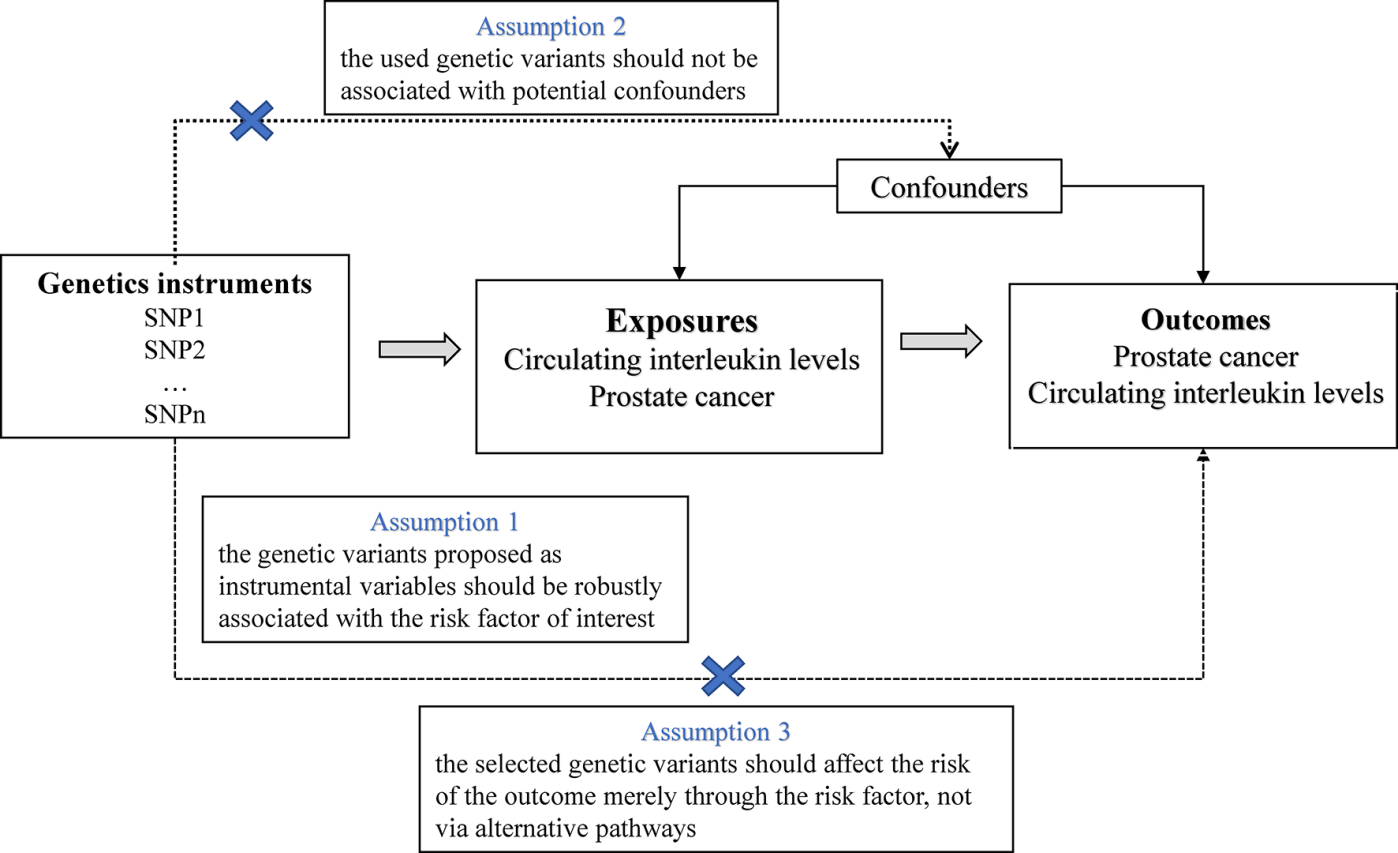
Overview and assumptions of the Mendelian randomization study design.

In this study, we used publicly published summary-level genetic data on ILs and prostate cancer from available genome-wide association studies (GWASs) (**Supplementary Table 1**). The MR approach in this study was on the strength of the following assumptions: (1) the selected IVs, that is, single nucleotide polymorphisms (SNPs) were strongly associated with the exposure; (2) the SNPs were not associated with confounding factors that bias the association of exposure with outcome; (3) and the SNPs affected the outcome exclusively *via* their effects on the selected risk factor (no horizontal pleiotropy) (15) (**Figure 1**). Of these, the second and third assumptions are collectively referred to as the independence of horizontal pleiotropy and can be examined using a range of statistical methods (16). We conducted a two-sample MR analysis to investigate the effects of ILs on prostate cancer risk using summary-level data. For ILs nominally related with prostate cancer (*P*<0.05), we conducted reverse MR analyses to verify whether genetically predicted prostate cancer affect the levels of these ILs.

### 2.2 Data sources for interleukins

Independent SNPs were selected as IVs if they had been associated with interleukins (*P* < 5×10^-8^) or prostate cancer (*P* < 5×10^-8^) at the genome-wide significance level in the GWAS source studies and in pair-wise linkage disequilibrium with the distance of 10000 kb and *r^2^* < 0.001 referring to the European population. We obtained SNPs robustly associated with ILs from existing GWASs (17–19). For IL-2, IL-4, IL-12, IL-23 and other ILs, none SNPs was chosen because they had not been associated with ILs at the genome-wide significance level (*P* < 5×10^-8^). After excluding those SNPs in linkage disequilibrium (LD) (*r^2^* >0.001 and clump window <10000 kb), 1 SNP was chosen as a IV for IL-1β from a GWAS comprising up to 13,577 participants of European descent (17). In the same way, 4 SNPs of IL-1ra, 4 SNPs of IL-6ra, 2 SNPs of IL-6, 2 SNPs of IL-8, 8 SNPs of IL-16, 8 SNPs of IL-18 and 11 SNPs of IL-27 were served as IVs from a GWAS with exceed 30,000 participants of European ancestry (18). For IL-17and IL-2 receptor alpha subunit (IL-2ra), one SNP each was used for the genetic association data from a GWAS containing up to 8,293 participants of European descent (19). Summary-level genetic data for these ILs were also acquired from the corresponding GWASs above. Details of the obtained SNPs for ILs are shown in **Supplementary Table 2**.

### 2.3 Data sources for prostate cancer

Genetic information for prostate cancer was acquired from three independent GWASs consortia for primary and replication analysis. Three datasets, including PRACTICAL, UK Biobank and the FinnGen consortium (Release 7), were used to extract the summary-level statistics of the associations between IL-related SNPs and prostate cancer. The PRACTICAL consortium currently does not include UK Biobank data and included 79,148 prostate cancer cases and 61,106 controls, all of which are white European ancestry. In the UK Biobank, 7,691 prostate cancer cases and 169,762 controls of European participants were included. Prostate cancer cases were defined according to self-reported archive codes, the International Classification of Diseases (ICD) 9 and 10, treatment/drug, and the office of population census and surveys from the UK Biobank study. The GWAS was conducted using logistic regression with the adjustment of age and the first 10 genetic principal components. The Release 7 released by the FinnGen consortium included 10,414 prostate cancer cases, and up to 124,994 controls. In FinnGen, prostate cancer cases were defined according to ICD-8, -9 and -10 codes with diagnostic information from nationwide registries. The GWAS analysis was adjusted for age of recruitment, top 10 principal components and recessive associations. Our analysis only included overall prostate cancer, which can be classified into several clinically relevant strata (e.g., T1, T2, T3, and M1) based on the Gleason score and prostate specific antigen.

Genetic IVs strongly associated with prostate cancer at the genome-wide significance level (*P*<5×10^-8^) were obtained from PRACTICAL, UK Biobank and the FinnGen consortium (R7) GWAS analysis on prostate cancer in European populations. After pruning SNPs in LD (*r^2^* >0.001 and clump window <10000 kb), 109, 31 and 88 SNPs were chosen as IVs used in the reverse MR analyses (**Supplementary Table 3**).

### 2.4 Instruments selection

**Figure 2:**
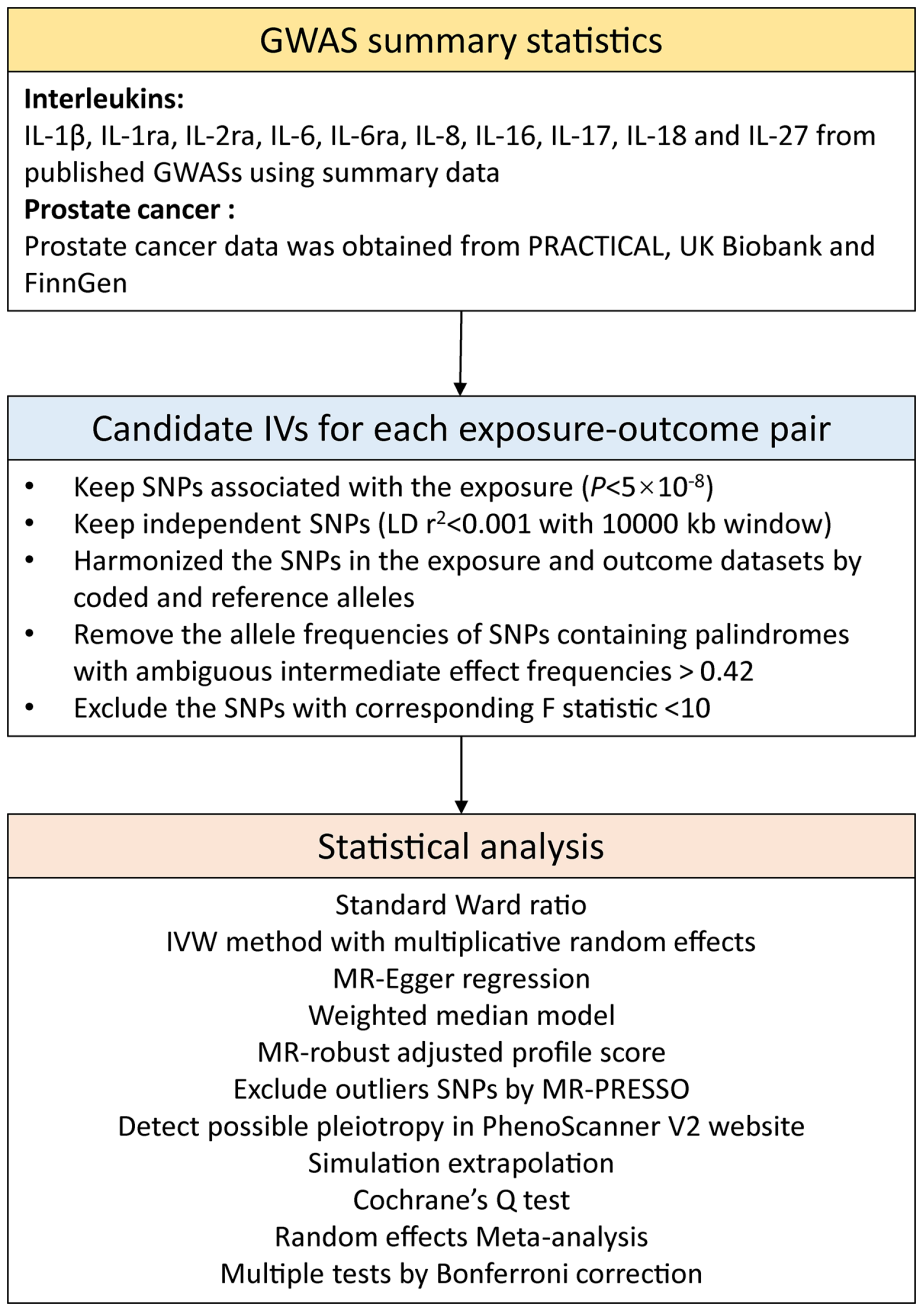
Study flame chart of the Mendelian randomization study.

The F statistic was computed to evaluate the strength of IVs (**Supplementary Table 4**). SNPs with F < 10 were considered as weak IVs and were discarded to ensure that all SNPs brought sufficient variance to the corresponding exposures (14). Power was estimated using a web tool (https://shiny.cnsgenomics.com/mRnd/) (**Supplementary Table 4**) (20). Unavailable IVs in the outcome data were replaced by proxy SNPs with high LD (r^2^ ≥0.8). Harmonization was then performed to calibrate exposed alleles and outcome SNPs, and alleles with intermediate effect frequencies (EAF > 0.42) or SNPs with incompatible alleles were discarded. The study frame chart is presented in **Figure 2**.

### 2.5 Statistical analysis

For ILs proxied by 1 SNP, the standard Ward ratio method was used to estimate the associations between ILs and prostate cancer. For the MR analysis based on ≥2 SNPs, the inverse variance weighted method with multiplicative random effects was used (13, 20). Estimates based on PRACTICAL, UK Biobank and FinnGen data for every IL were pooled using the random-effects meta-analysis method (21). Several sensitivity analyses, comprising the weighted median, MR-Egger, MR-robust adjusted profile score (MR-RAPS) and MR pleiotropy residual sum and outlier (MR-PRESSO) methods, were performed to assess the consistency of results and horizontal pleiotropy (20, 22–25). The weighted median method could generate consistent causal estimates if more than half of used IVs are valid (25). The MR-Egger method was conducted, which can adjust for bias from directional pleiotropic effects (23). The validity for MR-Egger method was also evaluated using the regression dilution *I* ^2^_GX_ statistic (26). If *I* ^2^_GX_ was lower than 0.9, we employed simulation extrapolation (SIMEX) to adjust for the dilution bias (26). MR-RAPS will be submitted as the square of the mean with less error if the instrument strength independent of the direct effects assumption is refined (24). MR-PRESSO analysis can detect possible outlying SNPs and provide causal estimates after the removal of outliers (27). Cochrane’s Q value was used to evaluate the heterogeneity among estimates of SNPs in one analysis (28). We scanned ILs-related SNPs associated traits at the genome-wide significance level in PhenoScanner V2 website (http://www.phenoscanner.medschl.cam.ac.uk/) to detect possible pleiotropy. Once the SNPs were associated with these potential confounders at the threshold of *P* < 5 × 10^-8^, a sensitivity analysis was performed after dropping these SNPs to validate the robustness of the results. Multiple tests were interpreted by Bonferroni correction. *P* value <0.005 (0.05/10 ILs) was recognized as significant association, and *P* value between <0.05 and ≥0.005 was deemed as suggestive associations. All statistical analyses were performed in R (version 4.0.1) with MendelianRandomization (version 0.4.2), TwoSampleMR (version 0.5.5) and MRPRESSO (version 1.0) packages.

## 3 Results

### 3.1 Forward MR analysis

**Figure 3:**
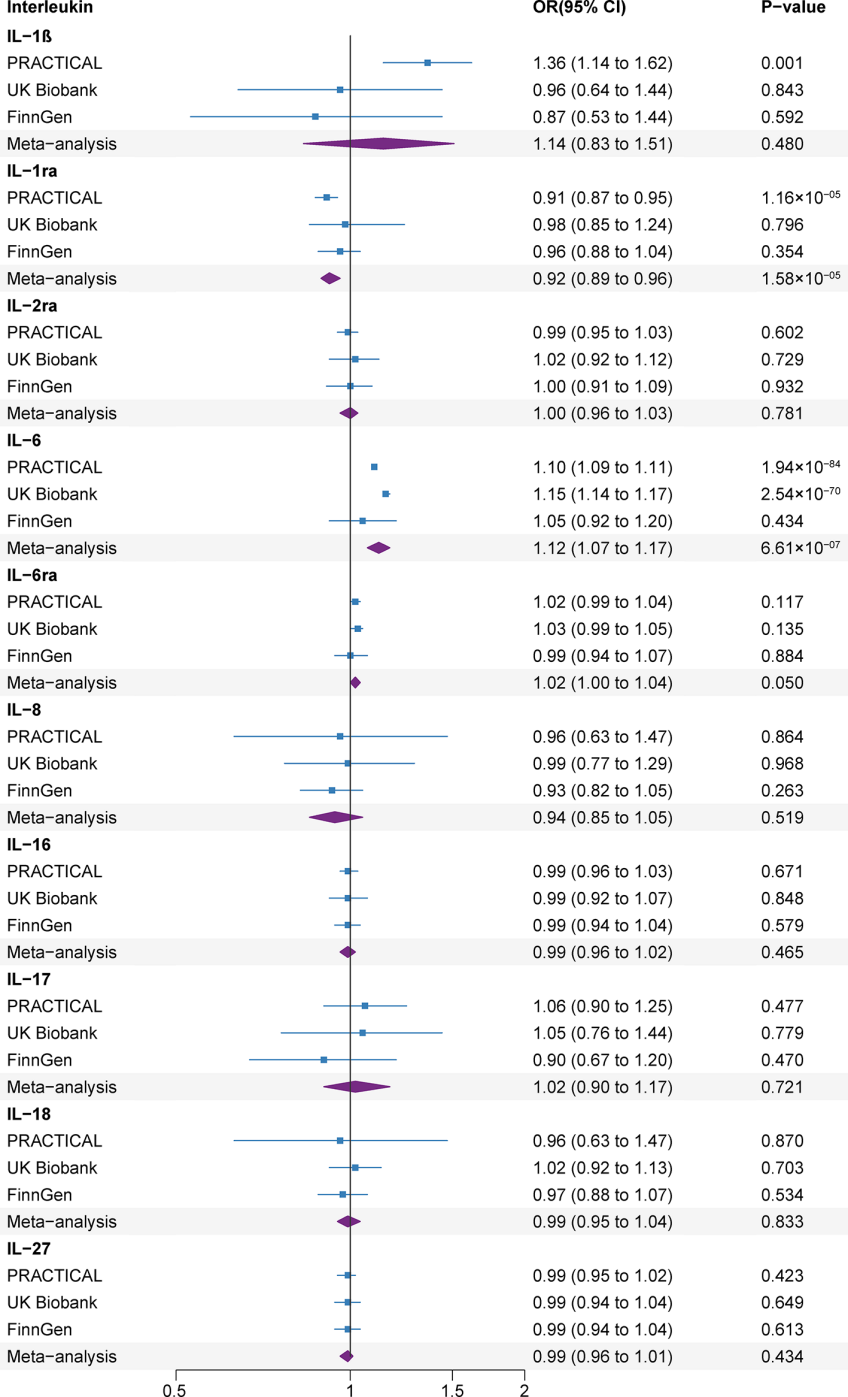
Associations of genetically predicted circulating interleukin levels with rheumatoid arthritis. CI, confidence interval; IL, interleukin; IL-1b, IL-1 beta; IL-1ra, IL-1 receptor antagonist; IL-2ra, IL-2 receptor alpha subunit; IL-6ra, IL-6 receptor subunit alpha; OR, odds ratio.

The F statistics for all IVs were greater than 10 (**Supplementary Table 4**). Genetic predisposition to the levels of 2 out of 10 ILs were related with the risk of prostate cancer at that Bonferroni-corrected significance level (**Figure 3**). The odds ratios (ORs) of prostate cancer were 0.92 (95% confidence interval (CI), 0.89, 0.96; *P=*1.58×10^-05^), and 1.12 (95% CI, 1.07, 1.17; *P=*6.61×10^-07^) for one standard deviation (SD) increase in genetic susceptibility to IL-1ra, and IL-6 levels, respectively. Genetic predisposition to levels of the other ILs were not related with the prostate cancer risk. To be specific, there were no associations of genetic predisposition to levels of IL-1ß (OR, 1.14, 95% CI, 0.83, 1.51; *P*=0.480), IL-2a (OR, 1.00, 95% CI, 0.96, 1.03; *P*=0.781), IL-6ra (OR, 1.02, 95% CI, 1.00, 1.04; *P*=0.050), IL-8 (OR, 0.94, 95% CI, 0.85, 1.05; *P*=0.519), IL-16 (OR, 0.99, 95% CI, 0.96, 1.02; *P*=0.465), IL-17 (OR, 1.02, 95% CI, 0.90, 1.17; *P*=0.721), IL-18 (OR, 0.99, 95% CI, 0.95, 1.04; *P*=0.833) and IL-27 (OR, 0.99, 95% CI, 0.96, 1.01; *P*=0.434) with prostate cancer risk.

The associations were consistent in the sensitivity analyses (**Supplementary Table 5**). We detected moderate heterogeneity in the analyses of IL-1ra in the UK Biobank and IL-6ra in the FinnGen, IL-8 and IL-27 in the PRACTICAL datasets (**Supplementary Table 6**). The MR-Egger intercept test did not detect the indications of horizontal pleiotropy (*P*>0.1) and MR-PRESSO analyses did not detect any outliers (**Supplementary Table 7**).

**Figure 4:**
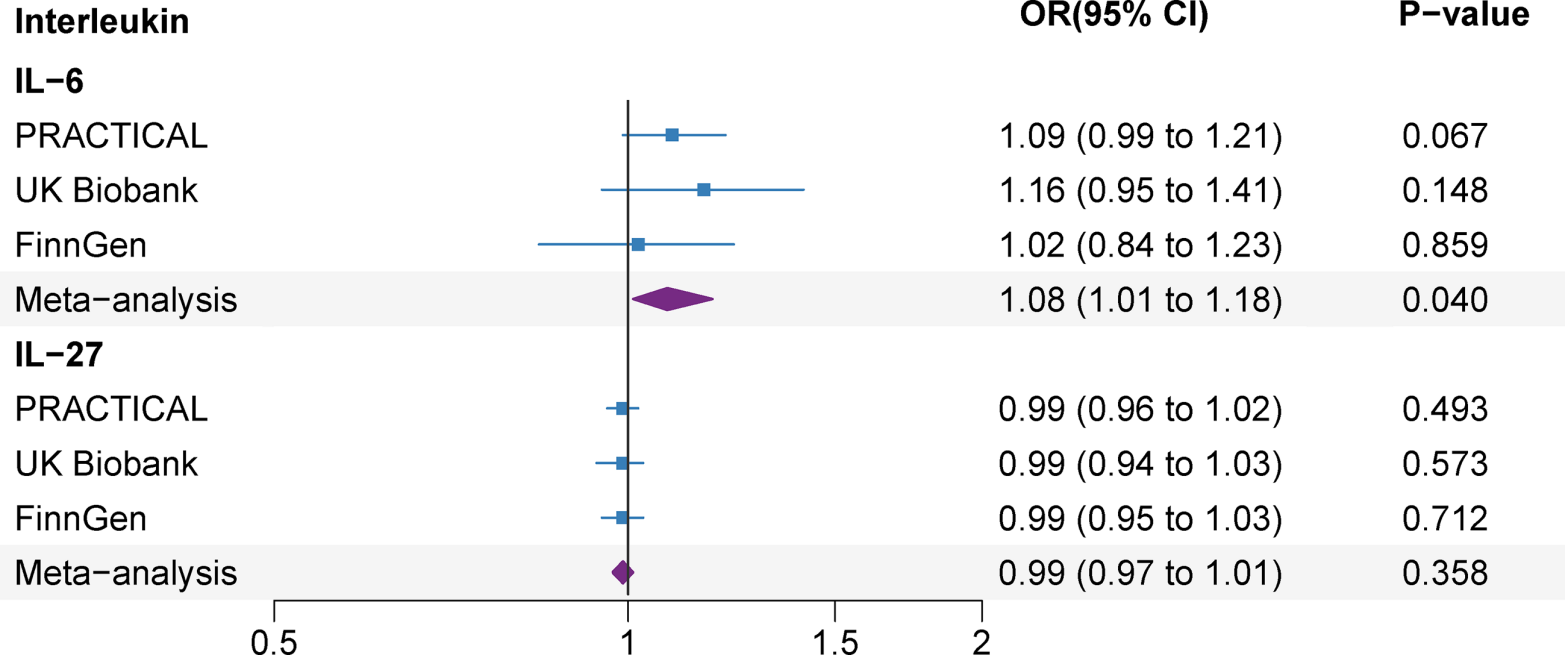
Associations of genetically predicted circulating interleukin levels with prostate cancer after the exception for body mass, smoking and high cholesterol. IL, interleukin; OR, odds ratio; CI, confidence interval.

Several SNPs (rs12126142 for IL-6ra, rs4959106 for IL-6, rs6734238 for IL-1ra, and rs10774624 for IL-27) were related with other phenotypes at the genome-wide significance level, containing smoking, height, body mass, high cholesterol, different fibrinogen levels, white blood cells and other autoimmune diseases (**Supplementary Table 8**). With exception for smoking (rs10774624 for IL-27) (29), body mass (rs10774624 for IL-27 and rs4959106 for IL-6) (30) and high cholesterol (rs4959106 for IL-6) (31), it was unlikely that other traits had pleiotropic effects on the observed associations between genetic predisposition to IL levels and prostate cancer risk. After excluding these potential pleiotropic SNPs, the associations between IL-6, IL-27 and prostate cancer risk remained consistent in the sensitivity analyses (**Figure 4** and **Supplementary Table 9**).

### 3.2 Reverse MR analysis

**Figure 5:**
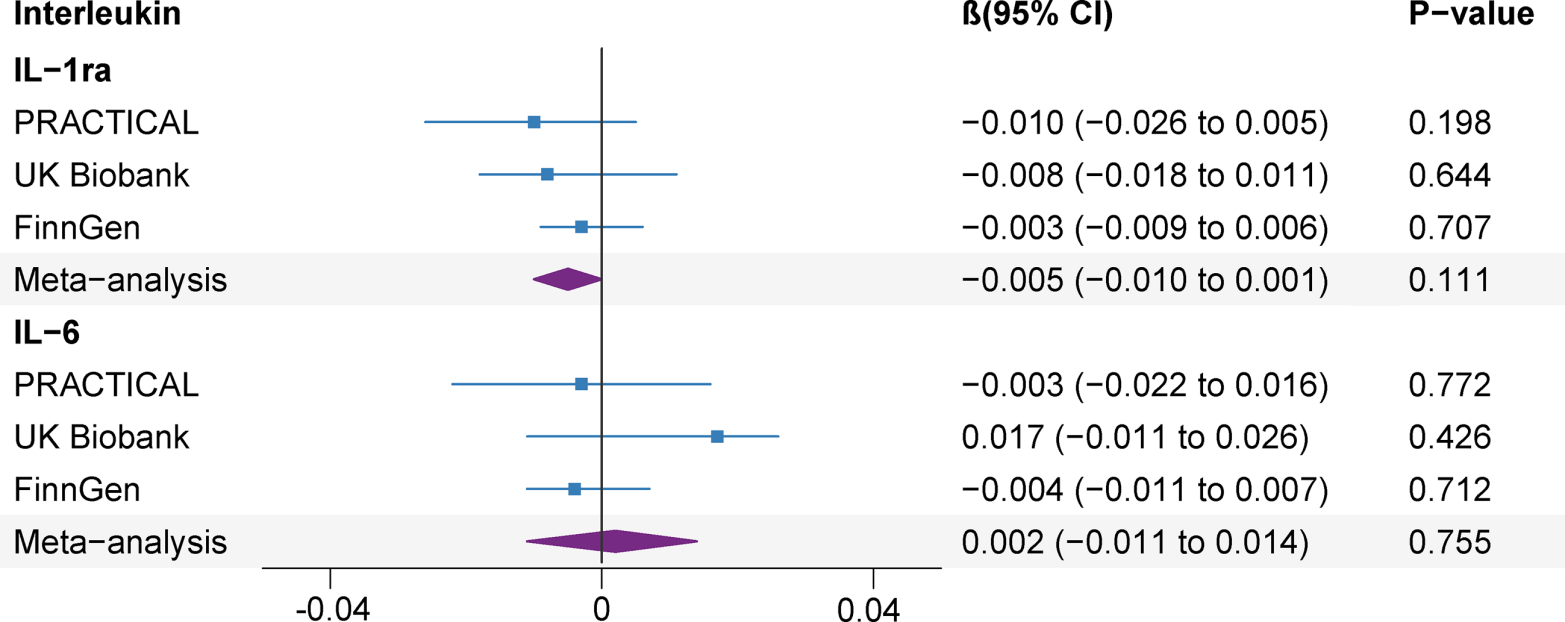
Association of genetic liability to prostate cancer with interleukin levels. CI, confidence interval; IL, interleukin; IL-1ra, IL-1 receptor antagonist.

Genetically predicted prostate cancer found no associations with studied ILs after Bonferroni multiple testing correction (IL-1ra and IL-6) (**Figure 5**). There was no association between genetic liability to prostate cancer and IL-1ra (β -0.005; 95% CI, -0.010, 0.001; *P*=0.111). The association remained consistent in the sensitivity analyses (**Supplementary Table 10**). Besides, genetically predicted prostate cancer was not associated with IL-6 (β 0.002; 95% CI, -0.011, 0.014; *P*=0.755) levels in the meta-analysis and sensitivity analyses (**Figure 5** and **Supplementary Table 10**). We detected low heterogeneity and no horizontal pleiotropy (*P* value for MR-Egger intercept or SIMEX >0.05) in these analyses (**Supplementary Table 11**).

## 4 Discussion

Our MR study found that genetic liability to IL-1ra and IL-6 levels were associated with the risk of prostate cancer. However, reverse MR analyses showed that genetically predicted prostate cancer was not associated with the higher levels of IL-1ra and IL-6.

The role of IL-1 signaling in the risk of development of prostate cancer has been controversial in previous observational studies. In a case-control study, individuals carrying the IL-1β (rs16944 and rs1143627) AG genotype were related with a lowered risk of prostate cancer as determined by real-time polymerase chain reaction of blood samples from 71 prostate cancer cases and 76 controls (32). This association was mainly observed in serum after diagnosis and the result suggests that the relationship between IL-1β and prostate cancer risk detected in case-control studies may be biased by reverse causation. Our study, which used MR techniques and data from three independent populations, did not detect such a potential causal relationship. Regarding IL-1ra, our findings are consistent with the results of a case-control study conducted in the San Antonio Center for Biomarkers of Risk of Prostate Cancer, which included a total of 123 prostate cancer patients and 127 age-matched controls (9). Pre-diagnostic serum concentrations were measured with the use of LabMAP technology in samples obtained at baseline (9). Before and after the adjustment for the Prostate Cancer Prevention Trial risk score, univariable and multivariable conditional logistic regression analysis showed that IL-1ra was significantly under-expressed in prostate cancer cases (9). Our study used a two-sample MR method and reinforced the potential role of IL-1ra in prostate cancer risk, although the association was mainly found in the PRACTICA population.

We observed that genetic predisposition to IL-6 levels were positively related with prostate cancer risk, and the result was consistent with previous observational studies (7, 8, 33). A meta-analysis indicated a significant association of the IL-6 gene rs1800795 and rs1800796 polymorphism with an overall elevated risk of prostate cancer based on the information collected from 118 GWAS studies consisting of 50,053 cases and 65,204 control samples (33). In another study, significant differences between prostate cancer patients and controls were found for IL-6 in the serum samples of 79 men (7). In addition, IL-6 levels were significantly higher in individuals with high-risk prostate cancer (7). Moreover, a case-control study found a positive association between IL-6 levels and prostate cancer risk in normal weight men with 353 cases and 696 controls (8).

The association between IL-8 and prostate cancer was not consistent in observational studies. Our research finding was consistent with results from one case-control study with 135 patients where IL-8 levels did not correlate to the diagnosis or aggressiveness of prostate cancer compared to controls (10). However, a meta-analysis including 6 case-control studies with up to 1,752 cases and 1,982 controls found IL-8 rs4073 polymorphism was associated with slightly higher prostate cancer risk (34). Our study employed more SNPs and samples and found the null association of IL-8 in prostate cancer risk.

Two studies did not detect an association between IL-16 and prostate cancer risk, which were consistent with our study. The MassARRAY technique was used to detect the genotypes of five cytokine gene SNPs in the blood samples of 90 prostate cancer patients and 140 control subjects in central China (11). Their study suggested that cytokine gene polymorphisms (IL-16 rs11556218 and rs7175701) might not be risk factors for prostate cancer in the central Chinese population (11). Meanwhile, a study evaluated the relationship between pre-diagnostic IL-16 serum levels and prostate cancer risk in 932 Caucasian cases and 942 controls in the Prostate, Lung, Colorectal, and Ovarian Cancer Screening Trial (12). No overall association between IL-16 and prostate cancer was detected in Caucasians (12).

Few studies focused on the associations between other ILs and prostate cancer risk, so our findings are novel and need to be validated. Reverse MR analysis detected that genetically predicted prostate cancer was not associated with levels of IL-1ra and IL-6, suggesting that prostate cancer itself might not upregulate these ILs.

The roles of IL-1 and IL-6 in the pathogenesis of prostate cancer have been proposed. Upon binding of IL-1a and IL-1ß to IL-1 receptor of type I, IL-1 receptor-related kinase 1 and TNF receptor-related factor 6 are recruited to the cytoplasmic domain of the receptor and transmit signals leading to activation of nuclear factor-kappaB (NF-kB). Interleukin-1 receptor antagonist gene attenuates IL-1a and IL-1ß induced signaling. IL-1a and IL-1ß activate NF-kB to promote tumor cell survival through anti-apoptotic signaling pathway in prostate cancer (35). Moreover, NF-kB can facilitate cell proliferation by inducing cyclin D1 and cyclin D2. Besides, loss of immune-expression of IL-1 receptor antagonist gene was a characteristic of prostate cancer compared to samples of normal prostate (36). The first possibility that IL-6 might be associated with prostate cancer progression comes from the discovery that the amount of circulating IL-6 is associated with hormone-refractory or metastatic prostate cancer (37–39). Several studies have shown that IL-6 and its downstream transcription factor STAT3 have been identified as core mediators involved in several steps of prostate tumor progression, including tumor initiation, tumor growth regulation and promotion of tumor metastasis (40–42).

A previous MR study investigated the association between ILs (IL-16, IL-18, IL-1a, IL-1ra, IL-2ra, IL-6, IL-7, IL-8, IL-12) and prostate cancer risk, and found no associations between ILs and prostate cancer risk. However, in that study, IVs were chosen at the genome-wide significance level with *P* value <1×10^-4^. In addition, the data of prostate cancer was derived from PRACTICAL consortium only. Compared with the previous MR study (43), our study has several strengths, including a comprehensive investigation of 10 ILs proxied by IVs with appropriate strength, a huge number of prostate cancer cases, reverse MR analyses, and replication in three independent population.

Using the MR design, this study was essentially free of reverse causality and residual confounding. We employed a range of methods to verify any violation of MR assumptions. The consistent direction and similar magnitude among the different MR models confirm the robustness of our MR estimates. We used the replication to further support the causal effect of IL-1ra and IL-6 on prostate cancer. Although the UK Biobank and FinnGen consortium estimates were different in replicate analyses, the consistent direction of the effect estimates was reassuring because they did not appear to have occurred by chance alone. Further meta-analysis showed that IL-1ra and IL-6 remained significant effects on prostate cancer. Anyhow, these two GWAS studies were different in gene chip, composition of population, quality control and data analysis, which may cause heterogeneity. The relationship between different populations requires further investigation.

Limitations must be addressed when interpreting these results. Although some sensitivity analyses were conducted and the causal associations in these analyses retained consistent, horizontal pleiotropy may be a problem that hinders causal inference, particularly for ILs that is proxied by several SNPs. However, no obvious evidence of horizontal pleiotropy was investigated using complementary statistical methods. By examining the potential pleiotropic effects of IL-related SNPs in PhenoScanner V2, the effects of rs10774624 for IL-27and rs4959106 for IL-6 on smoking, body weight, and high cholesterol may contribute to genetic predisposition to IL levels and prostate cancer. The positive relationship between IL-6 and prostate cancer and the null finding for IL-27 might be robust as the relationship retained consistent across analyses that excluded pleiotropic SNPs. The population of this study was European ancestry. While this can uttermost minimize population structure bias, when more GWAS data from other populations are publicly published, the generalizability of MR results needs to be further verified in future studies (44). The sample sizes of interleukin GWASs were relatively small, which may have a lower power to find enough related variants as IV. For some analyses of IL instrumented by SNPs, statistical power may be not sufficient to explain small phenotypic variance in rare cases. There may be interactions among ILs on prostate cancer development. Whereas, these interactions cannot be evaluated in current MR analyses based on summary-level data. We did not have access to publicly available GWASs for different types of prostate cancer, making it difficult to infer the differential effect of prostate cancer type on the causal relationship between ILs and prostate cancer.

## 5 Conclusions

In conclusion, this MR study suggests that ILs might influence the risk of prostate cancer in a causal way. Specifically, our MR study suggests that long-term IL-6 may increase the risk of prostate cancer and IL-1ra may reduce it. Our results indicated that more research studies are required to answer the connective link between ILs and prostate cancer.

## Data availability statement

The original contributions presented in the study are included in the article/**Supplementary Material**. Further inquiries can be directed to the corresponding authors.

## Ethics statement

All studies had been approved by a relevant ethical review board and participants had given informed consent. Ethical approval was not required because of the public characteristics of the data of GWAS.

## Author contributions

X-HW, Y-BW. and B-HL designed the research. B-HL and S-YY acquired and analyzed data. B-HL drafted the manuscript. X-HW, Y-BW, X-TZ, L-SL and B-HL interpreted data and made critical revisions of the manuscript. All authors contributed to the article and approved the submitted version.
