# Supplementary material for: Ten interleukins and risk of prostate cancer
Source: Front Oncol. 2023 Jan 17;13:1108633. doi: 10.3389/fonc.2023.1108633 (PMC9887118; doi:10.3389/fonc.2023.1108633)
Supplement: Supplementary file 1 [file DataSheet_1.pdf]

Supporting information

Ten interleukins and risk of prostate cancer

Supplementary Table 1. Included studies and consortium

Supplementary Table 2. Genetic instruments for circulating interleukins

Supplementary Table 3. Genetic instruments for prostate cancer

Supplementary Table 4. F statistics and power estimation

Supplementary Table 5. Associations of genetically predicted interleukins' levels with prostate cancer in sensitivity analyses

Supplementary Table 6. I^2_{GX}, MR-Egger intercept, Simulation extrapolation (SIMEX) for genetically predicted interleukins' levels with prostate cancer

Supplementary Table 7. The results of MR-PRESSO

Supplementary Table 8. Traits associated with ILs-associated SNPs in PhenoScanner V2 at the genome-wide significance level (P<5×10^{-8})

Supplementary Table 9. Associations of genetically predicted interleukins' levels with prostate cancer after exception for body mass, smoking and high cholesterol

Supplementary Table 10. Associations of genetically predicted prostate cancer with interleukins' levels in sensitivity analyses

Supplementary Table 11. I^2_{GX}, MR-Egger intercept, Simulation extrapolation (SIMEX) for genetically predicted prostate cancer with interleukins' levels

Supplementary Table 1. Included studies and consortium

IL / RA	Instruments	Source	Participants	Adjustments
IL-1β	1	Sliz et al GWAS	Up to 13,577 individuals of European ancestry	Age, sex, body mass index and the 10 first genetic principal components
IL-1ra<br>IL-6<br>IL-6ra<br>IL-8<br>IL-16<br>IL-18<br>IL-27	4<br>2<br>4<br>2<br>8<br>8<br>11	Folkersen et al GWAS	Over 30,000 individuals of European descent	Population structure and study-specific parameters
IL-2ra<br>IL-17	1<br>1	Ahola-Olli et al GWAS	Up to 8,293 individuals of European ancestry	Age, sex, body mass index, and the first ten genetic principal components
Prostate cancer	-	PRACTICAL	79,148 cases and 61,106 controls from of European populations	5-10 principal components
Prostate cancer	-	UK Biobank	7,691 cases and 169,762 controls of European descent	Age, sex, and the first 10 genetic principal components
Prostate cancer	-	FinnGen	10,414 cases and 124,994 controls of European descent	Age, sex, 10 genetic principal components, and genotyping batch

GWAS, genome-wide association studies; IL, interleukin; IL-1ra, IL-1 receptor antagonist; IL-2ra, IL-2 receptor alpha subunit; IL-6ra, IL-6 receptor subunit alpha.

Supplementary Table 2. Genetic instruments for circulating interleukins

IL	SNP	Chromosome	Position	Gene	EA	NEA	EAF	Beta	SE	P
IL-1β	rs6917603	6	30017071	*HLA locus*	T	C	0.70	0.163	0.023	1.76E-12
IL-1ra	rs4848312	2	113795197	*IL36B*	A	G	0.30	0.140	0.011	1.00E-37
IL-1ra	rs149418433	17	38159093	*PSMD3*	A	AT	0.38	0.065	0.011	4.30E-09
IL-1ra	rs6734238	2	113841030	*IL1F10*	A	G	0.61	0.200	0.010	2.50E-85
IL-1ra	rs62143194	19	54319624	*NLRP12*	C	G	0.78	-0.220	0.013	5.50E-63
IL-2ra	rs12722497	10	6095928	*IL2RA*	A	C	0.14	0.628	0.049	1.57E-38
IL-6	rs4959106	6	32583159	*HLA-DQA1*	T	C	0.54	-0.082	0.014	2.40E-09
IL-6	rs2228145	1	154426970	*IL6R*	A	C	0.62	-0.170	0.012	3.30E-45
IL-6ra	rs12126142	1	154425456	*IL6R*	A	G	0.39	0.960	0.007	3.40E-4116
IL-6ra	rs4845661	1	154673233	*KCNN3*	C	G	0.91	-0.120	0.016	6.60E-13
IL-6ra	rs36106412	1	154100693	*NUP210L*	A	G	0.14	0.092	0.016	1.70E-08
IL-6ra	rs7258728	19	41408746	*CYP2A7*	C	A	0.97	-0.290	0.052	2.40E-08
IL-8	rs972492	5	37903927	*GDNF-AS1*	A	C	0.14	-0.110	0.020	3.80E-08
IL-8	rs7655660	4	74589597	*CXCL8*	A	G	0.07	-0.190	0.024	2.70E-15
IL-16	rs58623354	3	141550696	*GRK7*	T	G	0.21	-0.078	0.014	4.80E-08
IL-16	rs117089718	9	20228466	*MLLT3*	T	C	0.97	-0.220	0.034	2.00E-10
IL-16	rs774284803	15	84483728	*ADAMTSL3*	A	G	0.03	-0.840	0.130	3.60E-11
IL-16	rs4778639	15	81600451	*IL16*	T	G	0.92	0.960	0.018	6.20E-621
IL-16	rs150568717	15	86537235	*AGBL1*	T	C	0.97	0.800	0.120	3.90E-11
IL-16	rs859	15	81601322	*IL16*	A	G	0.73	0.140	0.012	4.40E-33
IL-16	rs1564641	15	81790468	*STARD5*	A	G	0.48	0.067	0.011	1.20E-09
IL-16	rs10424405	19	54321933	*NLRP12*	A	G	0.78	-0.120	0.013	5.30E-21
IL-17	rs1530455	3	122854899	*PDIA5*	T	C	0.60	0.108	0.017	4.71E-10
IL-18	rs17229943	5	68682536	*RAD17*	A	C	0.94	-0.180	0.028	6.40E-11
IL-18	rs916964	7	26146063	*NFE2L3*	C	G	0.80	0.074	0.013	6.40E-09
IL-18	rs385076	2	32489851	*NLRC4*	T	C	0.37	-0.180	0.012	3.20E-55
IL-18	rs9867857	3	156491160	*LEKR1*	T	C	0.47	0.060	0.010	5.10E-09
IL-18	rs4606077	8	144656754	*NAPRT*	T	C	0.24	-0.088	0.012	4.60E-13
IL-18	rs5002812	11	105036178	*CARD18*	A	G	0.74	-0.078	0.012	5.60E-10
IL-18	rs5744249	11	112025306	*IL18*	A	C	0.76	0.220	0.012	3.70E-74
IL-18	rs10424405	19	54321933	*NLRP12*	A	G	0.78	-0.240	0.013	1.20E-73
IL-27	rs1257169	2	134963862	*MGAT5*	A	C	0.53	-0.097	0.011	1.30E-17
IL-27	rs9715769	4	38774489	*TLR10*	A	C	0.82	-0.096	0.015	4.50E-11
IL-27	rs11711157	3	194061826	*CPN2*	T	C	0.29	-0.090	0.014	7.60E-11
IL-27	rs966541	12	29491528	*ERGIC2*	A	G	0.72	-0.062	0.011	3.20E-08
IL-27	rs111072793	17	7079593	*ASGR1*	A	C	0.20	0.098	0.015	2.90E-11

IL-27	rs704	17	26694861	*VTN*	A	G	0.47	-0.130	0.010	1.40E-39
IL-27	rs59975010	19	12497383	*ZNF799*	T	C	0.76	-0.084	0.014	5.40E-09
IL-27	rs12625762	20	34158587	*ERGIC3*	A	G	0.14	0.082	0.015	3.20E-08
IL-27	rs11599750	10	101805442	*CPN1*	T	C	0.38	-0.180	0.010	8.00E-66
IL-27	rs10774624	12	111833788	*SH2B3*	A	G	0.53	-0.076	0.010	2.20E-13
IL-27	rs4905	19	4237067	*EBI3*	A	G	0.70	0.670	0.010	2.30E-938

EA, effect allele; EAF, effect allele frequency; IL, interleukin; IL-1ra, IL-1 receptor antagonist; IL-2ra, IL-2 receptor alpha subunit; IL-6ra, IL-6 receptor subunit alpha; NEA, non-effect allele; Position, position based on hg_37; SNP, single nucleotide polymorphism.

Supplementary Table 3. Genetic instruments for prostate cancer

Source	SNP	Chromosome	Position	EA	NEA	EAF	Beta	SE	P
PRACTICAL	rs1004030	14	23305649	T	C	0.5844	0.0462	0.0082	1.55E-08
PRACTICAL	rs10095746	8	128463486	T	C	0.7829	-0.0802	0.0102	3.32E-15
PRACTICAL	rs10124307	9	33803408	T	C	0.6276	-0.0453	0.0083	4.36E-08
PRACTICAL	rs10499617	7	40782572	T	C	0.857	0.0756	0.0128	3.56E-09
PRACTICAL	rs10807802	7	20444952	A	T	0.4933	-0.0472	0.0086	4.43E-08
PRACTICAL	rs11239315	10	45503571	T	C	0.9501	0.1012	0.0185	4.91E-08
PRACTICAL	rs11345065	8	23410965	A	C	0.7122	0.0622	0.0089	2.46E-12
PRACTICAL	rs114674839	5	1228166	T	C	0.045	0.1168	0.0197	3.32E-09
PRACTICAL	rs11585865	1	204027009	A	G	0.4606	-0.0457	0.008	1.29E-08
PRACTICAL	rs115880776	2	173258607	T	C	0.0195	-0.1861	0.031	2.00E-09
PRACTICAL	rs116349314	1	150248767	A	T	0.9457	-0.1023	0.0176	5.71E-09
PRACTICAL	rs11650424	17	435896	T	C	0.2376	0.057	0.0094	1.51E-09
PRACTICAL	rs11656715	17	7769228	A	C	0.8774	-0.0686	0.0123	2.51E-08
PRACTICAL	rs116789570	2	173298891	A	G	0.9691	0.2178	0.0243	3.13E-19
PRACTICAL	rs11681263	2	111868010	A	C	0.251	-0.062	0.0092	1.43E-11
PRACTICAL	rs11693159	2	242152658	A	G	0.2271	-0.0522	0.0096	4.65E-08
PRACTICAL	rs11695848	2	85692999	A	C	0.6308	0.0527	0.0087	1.14E-09
PRACTICAL	rs11781472	8	127857892	A	G	0.0351	-0.1433	0.0225	1.83E-10
PRACTICAL	rs117821438	17	47325867	A	T	0.9252	-0.1003	0.0149	1.50E-11
PRACTICAL	rs118005503	19	32167803	C	G	0.0889	-0.0902	0.0156	7.31E-09
PRACTICAL	rs1180779	7	97634876	A	C	0.5899	0.066	0.0082	1.05E-15
PRACTICAL	rs11876000	18	73035513	T	G	0.414	0.0439	0.008	3.99E-08
PRACTICAL	rs12193858	6	109277908	A	T	0.8723	-0.0673	0.012	2.21E-08
PRACTICAL	rs12203518	6	41510291	C	G	0.2869	0.0767	0.0088	1.94E-18
PRACTICAL	rs12416098	10	47540959	A	G	0.8153	-0.1031	0.0142	3.20E-13
PRACTICAL	rs12461874	19	17180358	A	C	0.2771	-0.0497	0.0091	4.85E-08
PRACTICAL	rs12476059	2	238351347	T	C	0.8733	-0.0694	0.0123	1.58E-08
PRACTICAL	rs12663441	6	11171163	T	C	0.1287	-0.0669	0.012	2.52E-08
PRACTICAL	rs12776659	10	834057	A	C	0.1693	0.0738	0.0109	1.07E-11
PRACTICAL	rs1283105	3	106962305	C	G	0.6213	-0.047	0.0082	8.95E-09
PRACTICAL	rs12944450	17	46546346	T	C	0.0372	0.1552	0.0237	6.26E-11

PRACTICAL	rs12974417	19	38725859	T	C	0.5847	0.0708	0.0083	1.33E-17
PRACTICAL	rs13099541	3	87090905	A	G	0.8572	-0.0768	0.0112	8.20E-12
PRACTICAL	rs13150559	4	105934084	T	C	0.8764	0.0693	0.0122	1.45E-08
PRACTICAL	rs13302748	9	19063108	T	C	0.5391	0.044	0.0079	2.94E-08
PRACTICAL	rs1370038	3	86966639	T	C	0.643	0.0463	0.0083	2.68E-08
PRACTICAL	rs139048524	3	170083585	T	C	0.0385	-0.1682	0.0228	1.81E-13
PRACTICAL	rs139283528	2	63938756	A	G	0.0149	-0.2055	0.0349	3.71E-09
PRACTICAL	rs142436749	3	169093100	A	G	0.9879	-0.2212	0.0378	4.70E-09
PRACTICAL	rs142449279	15	56406361	A	G	0.0221	-0.1763	0.0312	1.57E-08
PRACTICAL	rs143344195	8	128070599	A	G	0.988	0.2475	0.041	1.56E-09
PRACTICAL	rs143368544	8	128117736	T	C	0.0212	0.2339	0.0281	9.30E-17
PRACTICAL	rs147410223	8	23507379	A	G	0.6951	0.0549	0.0087	2.26E-10
PRACTICAL	rs1582874	3	141115219	T	C	0.5582	-0.0435	0.008	4.77E-08
PRACTICAL	rs17626629	7	47437072	A	T	0.5074	0.0439	0.008	3.73E-08
PRACTICAL	rs17885652	11	2167303	A	G	0.2428	0.0593	0.0093	1.92E-10
PRACTICAL	rs181069596	8	128330539	C	G	0.0081	0.8533	0.0442	3.94E-83
PRACTICAL	rs1821640	2	174227458	A	T	0.518	-0.0465	0.0084	2.87E-08
PRACTICAL	rs1881502	11	1507512	T	C	0.1903	0.0581	0.0101	7.42E-09
PRACTICAL	rs2002731	17	36088530	T	C	0.3771	0.1085	0.0084	7.86E-38
PRACTICAL	rs2058843	17	69056825	A	T	0.3994	0.0504	0.0081	4.81E-10
PRACTICAL	rs2165108	2	111861993	A	T	0.0458	0.1104	0.0189	4.81E-09
PRACTICAL	rs2187364	11	102392380	T	G	0.7787	-0.0558	0.0099	1.50E-08
PRACTICAL	rs2238774	22	19757339	T	C	0.5445	0.0443	0.0081	4.61E-08
PRACTICAL	rs2277283	11	61908440	T	C	0.6868	-0.0558	0.0089	3.03E-10
PRACTICAL	rs2483184	13	73701852	C	G	0.8825	-0.0694	0.0125	3.10E-08
PRACTICAL	rs2510802	4	95410168	A	G	0.7557	0.0542	0.0092	4.35E-09
PRACTICAL	rs2517534	6	31017334	A	G	0.4326	0.0458	0.008	1.14E-08
PRACTICAL	rs2529758	7	20851129	T	G	0.5328	-0.0464	0.008	6.53E-09
PRACTICAL	rs305457	1	88195672	A	G	0.8411	-0.0589	0.0107	3.80E-08
PRACTICAL	rs34314670	17	30080257	T	C	0.2305	-0.0603	0.0102	3.53E-09
PRACTICAL	rs34349762	18	56741676	T	C	0.6937	-0.0494	0.0086	7.99E-09
PRACTICAL	rs34560402	11	66872320	T	C	0.0482	0.1106	0.0197	2.01E-08
PRACTICAL	rs34578638	6	153358706	A	G	0.093	-0.0789	0.0142	3.10E-08

PRACTICAL	rs34983238	21	42868997	A	C	0.9006	0.1002	0.0146	6.12E-12
PRACTICAL	rs373745804	2	43502132	T	C	0.6438	-0.0646	0.0084	1.92E-14
PRACTICAL	rs375314312	11	47421965	T	G	0.5321	-0.0468	0.0085	3.37E-08
PRACTICAL	rs3861973	6	160628905	T	C	0.2717	-0.0674	0.0092	2.75E-13
PRACTICAL	rs41317513	1	150525425	A	G	0.0388	-0.1306	0.0227	9.17E-09
PRACTICAL	rs4699158	4	106065949	T	C	0.0887	0.0806	0.0137	3.83E-09
PRACTICAL	rs4713242	6	29718220	A	G	0.2722	0.0497	0.0088	1.92E-08
PRACTICAL	rs56031543	4	73699144	A	G	0.8326	0.0597	0.0108	3.69E-08
PRACTICAL	rs56073101	8	127949625	A	C	0.9759	0.1613	0.0269	2.07E-09
PRACTICAL	rs56237946	11	113548935	T	C	0.177	-0.0584	0.0106	3.53E-08
PRACTICAL	rs56721892	3	113287314	A	G	0.7788	0.0538	0.0097	2.74E-08
PRACTICAL	rs59710626	19	38548094	T	G	0.1353	-0.0679	0.0119	1.21E-08
PRACTICAL	rs62063875	17	69156750	T	C	0.0882	-0.0941	0.0142	3.70E-11
PRACTICAL	rs62106670	2	8597123	T	C	0.3791	0.0524	0.009	7.11E-09
PRACTICAL	rs62217467	21	42771554	A	G	0.0278	0.1438	0.0258	2.59E-08
PRACTICAL	rs62451083	7	27929316	T	C	0.7149	0.0603	0.0089	1.49E-11
PRACTICAL	rs6508285	18	51769949	C	G	0.444	0.0441	0.0081	4.67E-08
PRACTICAL	rs669017	6	117093340	T	C	0.7406	0.0755	0.0091	1.08E-16
PRACTICAL	rs6798592	3	112946985	C	G	0.2766	-0.0533	0.009	3.43E-09
PRACTICAL	rs71465417	11	68840490	T	C	0.0153	0.2224	0.0341	6.96E-11
PRACTICAL	rs7172947	15	40877322	A	G	0.8367	0.0613	0.011	2.22E-08
PRACTICAL	rs72985681	18	76761852	A	T	0.8058	0.0777	0.0107	3.78E-13
PRACTICAL	rs73025562	6	160562481	A	G	0.258	0.0514	0.009	1.24E-08
PRACTICAL	rs73425403	9	18551961	C	G	0.3022	-0.0489	0.0087	1.69E-08
PRACTICAL	rs74829122	5	1241565	A	G	0.9368	0.1127	0.0186	1.39E-09
PRACTICAL	rs7519417	1	204449952	T	C	0.2713	-0.0874	0.009	2.84E-22
PRACTICAL	rs7553195	1	154837796	T	C	0.5976	-0.0455	0.008	1.44E-08
PRACTICAL	rs7587923	2	85737997	T	C	0.2104	-0.0685	0.0099	4.39E-12
PRACTICAL	rs7598541	2	10700516	A	G	0.3876	-0.0562	0.0082	5.64E-12
PRACTICAL	rs76152355	11	69006249	T	C	0.9468	0.1066	0.0184	7.16E-09
PRACTICAL	rs78072686	8	127821880	T	C	0.9797	-0.2252	0.0273	1.49E-16
PRACTICAL	rs7867490	9	132549740	T	G	0.1876	0.0557	0.0101	3.32E-08
PRACTICAL	rs79223973	3	151992162	A	C	0.8967	0.0879	0.0135	7.45E-11

PRACTICAL	rs79709647	3	169689793	A	G	0.9331	0.0937	0.0168	2.66E-08
PRACTICAL	rs79766835	2	62710273	C	G	0.0938	0.1286	0.0137	8.42E-21
PRACTICAL	rs80126717	15	66587581	A	G	0.2465	0.0544	0.0094	7.18E-09
PRACTICAL	rs817864	9	110142648	A	G	0.2768	0.0549	0.0088	4.10E-10
PRACTICAL	rs823105	1	205657570	A	G	0.4535	-0.0435	0.008	4.53E-08
PRACTICAL	rs878987	11	134266372	A	G	0.8541	-0.0639	0.0117	4.77E-08
PRACTICAL	rs916880	7	27229119	A	G	0.9149	0.084	0.015	2.21E-08
PRACTICAL	rs9306894	2	20878105	A	G	0.6367	-0.0777	0.0082	1.92E-21
PRACTICAL	rs9443189	6	76495882	A	G	0.8571	0.0635	0.0116	4.68E-08
PRACTICAL	rs9457699	6	160071652	T	G	0.6965	0.0485	0.0088	3.34E-08
PRACTICAL	rs9625483	22	28888939	A	G	0.0288	0.1338	0.024	2.43E-08
PRACTICAL	rs9839080	3	127711997	T	C	0.4268	0.0564	0.008	2.24E-12
UK Biobank	rs1015521	4	106115450	T	G	0.3026	-0.1278	0.01822	2.35E-12
UK Biobank	rs10441523	8	127924563	T	C	0.6602	-0.1087	0.01838	3.34E-09
UK Biobank	rs10486567	7	27976563	A	G	0.2121	-0.1266	0.02016	3.41E-10
UK Biobank	rs10953238	7	97676259	T	C	0.4866	-0.0989	0.01703	6.37E-09
UK Biobank	rs11667256	19	38735804	T	A	0.4955	-0.1111	0.01703	6.97E-11
UK Biobank	rs139653137	12	53260717	A	G	0.0905	0.1768	0.03186	2.88E-08
UK Biobank	rs146569428	11	2199686	A	G	0.2225	0.1404	0.02145	5.87E-11
UK Biobank	rs1567669	8	23538533	G	A	0.6736	-0.1063	0.01858	1.06E-08
UK Biobank	rs17138476	17	36075605	T	C	0.1779	0.1331	0.02341	1.31E-08
UK Biobank	rs200470358	10	51462599	T	C	0.618	-0.1474	0.01977	9.06E-14
UK Biobank	rs2166689	8	128478315	T	G	0.78	-0.1662	0.02162	1.52E-14
UK Biobank	rs2739443	19	51341326	A	G	0.096	-0.1756	0.02752	1.79E-10
UK Biobank	rs28691002	17	7805353	T	C	0.07096	0.2058	0.03617	1.27E-08
UK Biobank	rs453875	8	128321411	A	G	0.4012	-0.1082	0.01728	3.88E-10
UK Biobank	rs530543670	5	1889321	A	G	0.3272	0.1293	0.01941	2.69E-11
UK Biobank	rs540136	6	117093645	A	G	0.7624	0.1073	0.01952	3.87E-08
UK Biobank	rs5759167	22	43500212	T	G	0.4654	-0.1411	0.01706	1.32E-16
UK Biobank	rs61436251	3	170083629	G	C	0.179	-0.1435	0.02147	2.30E-11
UK Biobank	rs6423444	20	62272411	A	G	0.2894	-0.1162	0.01876	5.82E-10
UK Biobank	rs6854173	4	95537849	G	A	0.4793	0.09379	0.01717	4.67E-08
UK Biobank	rs6992687	8	23458354	A	T	0.3973	-0.09724	0.01725	1.74E-08

UK Biobank	rs7127508	11	69014017	C	T	0.4337	0.1196	0.01746	7.21E-12
UK Biobank	rs72709458	5	1283755	T	C	0.1836	-0.1572	0.02147	2.45E-13
UK Biobank	rs72930627	11	68867121	A	C	0.169	0.1292	0.02362	4.58E-08
UK Biobank	rs7642887	3	87172632	C	T	0.4794	-0.09475	0.01711	3.06E-08
UK Biobank	rs76733736	2	173286319	A	T	0.04783	-0.2097	0.0379	3.16E-08
UK Biobank	rs77541621	8	128077146	A	G	0.04657	0.7109	0.05626	1.33E-36
UK Biobank	rs79766835	2	62710273	C	G	0.1156	0.1635	0.02862	1.10E-08
UK Biobank	rs8071558	17	69107673	G	C	0.5012	-0.1416	0.01708	1.13E-16
UK Biobank	rs9257566	6	29144532	T	C	0.1247	-0.1423	0.02463	7.53E-09
UK Biobank	rs9364554	6	160833664	T	C	0.3297	0.1097	0.01842	2.60E-09
FinnGen	rs10069690	5	1279675	T	C	0.296028	-0.1059	0.0167462	2.55E-10
FinnGen	rs10099034	8	127255093	T	C	0.245071	-0.125914	0.0179092	2.05E-12
FinnGen	rs10429276	8	127672086	G	T	0.101847	0.167453	0.0244452	7.38E-12
FinnGen	rs10505477	8	127395198	G	A	0.497493	-0.17998	0.0151076	1.01E-32
FinnGen	rs10788157	10	121264760	A	G	0.250764	-0.0980725	0.0177209	3.13E-08
FinnGen	rs10934228	3	113380614	G	C	0.453664	0.087353	0.0151692	8.48E-09
FinnGen	rs111689685	1	153874640	A	G	0.265126	0.0954995	0.0170158	2.00E-08
FinnGen	rs112676730	12	52864687	G	C	0.103297	0.1756	0.0241672	3.70E-13
FinnGen	rs113149952	17	49420386	A	G	0.0184111	0.422683	0.0509319	1.05E-16
FinnGen	rs115463731	2	241021333	G	A	0.0520903	-0.329394	0.0380234	4.60E-18
FinnGen	rs11658063	17	37743881	C	G	0.293797	-0.190365	0.0169556	3.00E-29
FinnGen	rs11672691	19	41479679	A	G	0.283718	-0.105152	0.017063	7.16E-10
FinnGen	rs116931900	17	48724952	T	G	0.0217794	0.672483	0.0442993	4.77E-52
FinnGen	rs117059295	8	129080253	C	T	0.028551	0.246777	0.0447944	3.61E-08
FinnGen	rs117191669	17	46842871	C	A	0.00747235	0.610373	0.0793761	1.48E-14
FinnGen	rs11754046	6	159817755	A	G	0.151052	0.116087	0.0207242	2.12E-08
FinnGen	rs11768309	7	98144500	A	C	0.476862	-0.0831421	0.0151493	4.06E-08
FinnGen	rs12152490	3	127891842	G	A	0.611837	0.108319	0.0163527	3.50E-11
FinnGen	rs12155172	7	20954872	G	A	0.780742	-0.119526	0.0183201	6.83E-11
FinnGen	rs12203518	6	41542553	C	G	0.351216	0.0993418	0.0158785	3.94E-10
FinnGen	rs1239176	17	38849878	C	T	0.466262	0.0827807	0.0151776	4.92E-08
FinnGen	rs12419346	11	2195881	C	G	0.203551	0.143595	0.0184103	6.20E-15
FinnGen	rs12680310	8	127920561	T	C	0.130144	0.147413	0.0219909	2.04E-11

FinnGen	rs12793759	11	69207088	A	G	0.0924812	0.242784	0.0246963	8.30E-23
FinnGen	rs13176243	5	1512901	T	C	0.0879371	-0.160148	0.0282194	1.39E-08
FinnGen	rs13245339	7	27522478	C	T	0.239903	-0.100412	0.0179186	2.10E-08
FinnGen	rs13249021	8	126912167	G	A	0.566314	-0.107507	0.0152783	1.97E-12
FinnGen	rs13260097	8	126648882	A	G	0.157985	0.117171	0.0206916	1.49E-08
FinnGen	rs13282582	8	127565038	T	C	0.0497795	-0.206437	0.0369888	2.39E-08
FinnGen	rs138249239	17	49230743	G	A	0.00978213	0.519323	0.0683313	2.96E-14
FinnGen	rs1396870	17	48418050	G	T	0.239989	0.097535	0.0175113	2.55E-08
FinnGen	rs140516285	17	47752651	C	G	0.0151093	0.461589	0.0576679	1.20E-15
FinnGen	rs141162615	8	126820558	C	G	0.0364704	-0.255782	0.0439325	5.81E-09
FinnGen	rs145127226	17	45903275	A	G	0.0136024	0.358435	0.0611982	4.71E-09
FinnGen	rs145315307	5	169477001	G	T	0.0368121	-0.255114	0.0437409	5.46E-09
FinnGen	rs145988452	14	37564370	C	T	0.0423225	-0.256418	0.0401725	1.74E-10
FinnGen	rs1465618	2	43326810	C	T	0.788683	-0.102253	0.0182951	2.28E-08
FinnGen	rs147940485	8	126560801	A	G	0.0125024	0.386048	0.0627438	7.61E-10
FinnGen	rs14983	11	102520694	A	G	0.24093	0.103756	0.0174758	2.90E-09
FinnGen	rs151265313	8	126402262	A	G	0.0501526	0.205153	0.0337323	1.19E-09
FinnGen	rs17002161	4	76677520	G	C	0.311989	0.0889185	0.0162665	4.59E-08
FinnGen	rs17138478	17	37713312	A	C	0.157623	0.117007	0.0205265	1.20E-08
FinnGen	rs182955053	8	125692489	C	A	0.0718294	0.169312	0.0285574	3.05E-09
FinnGen	rs1986719	17	47882630	T	C	0.974096	-0.330549	0.0444482	1.03E-13
FinnGen	rs200470358	10	46133223	A	G	0.674465	-0.111902	0.0163606	7.93E-12
FinnGen	rs2305073	2	241236604	C	T	0.353537	-0.092641	0.0160372	7.62E-09
FinnGen	rs2510773	4	94548157	A	G	0.276815	-0.0935481	0.017083	4.35E-08
FinnGen	rs2648868	8	128052562	A	G	0.0920723	0.141201	0.0255072	3.10E-08
FinnGen	rs2701111	12	114227115	A	G	0.495402	-0.0829474	0.0151812	4.66E-08
FinnGen	rs2739443	19	50838070	A	G	0.06487	-0.198102	0.031451	3.00E-10
FinnGen	rs2811477	3	128179940	T	C	0.100383	0.150794	0.0245254	7.82E-10
FinnGen	rs28435470	12	132490887	A	G	0.581906	-0.087425	0.0153709	1.29E-08
FinnGen	rs2928672	8	23552812	T	C	0.366194	-0.0874056	0.0158101	3.23E-08
FinnGen	rs34608985	17	46814178	G	T	0.0487794	0.23409	0.0341046	6.70E-12
FinnGen	rs35304108	8	127555760	A	G	0.0624006	0.441949	0.0299857	3.64E-49
FinnGen	rs3891696	8	126931070	T	G	0.119358	0.128041	0.0229341	2.36E-08

FinnGen	rs3999773	8	127463337	A	T	0.414347	-0.18677	0.0155565	3.31E-33
FinnGen	rs415304	20	63566883	A	G	0.542605	0.0964242	0.0153914	3.73E-10
FinnGen	rs4722677	7	27215212	T	A	0.91266	0.156335	0.0278786	2.05E-08
FinnGen	rs530543670	5	1889207	A	G	0.416871	0.138457	0.0154569	3.32E-19
FinnGen	rs55947550	17	48753032	C	G	0.187375	0.135985	0.0191871	1.37E-12
FinnGen	rs56257047	3	170270498	T	C	0.179146	-0.146204	0.0202875	5.74E-13
FinnGen	rs572014349	17	45591879	A	G	0.0407103	0.207892	0.0376468	3.35E-08
FinnGen	rs57287517	8	127556492	T	G	0.11123	0.194072	0.0236079	2.02E-16
FinnGen	rs62003539	14	52922981	C	T	0.138222	-0.127306	0.0225275	1.59E-08
FinnGen	rs62076101	17	47804877	A	G	0.0200772	0.388698	0.0493071	3.19E-15
FinnGen	rs62177748	2	62681742	T	C	0.147125	0.129812	0.0211016	7.66E-10
FinnGen	rs629849	6	160073377	G	A	0.90568	-0.161121	0.0250416	1.24E-10
FinnGen	rs630045	6	116878627	G	C	0.332565	-0.0958829	0.0161725	3.05E-09
FinnGen	rs6470454	8	126580796	G	A	0.834512	-0.131395	0.0205035	1.47E-10
FinnGen	rs6983515	8	127059677	G	A	0.797461	-0.161244	0.0185162	3.09E-18
FinnGen	rs6990480	8	127457463	A	G	0.91584	-0.279679	0.0263228	2.28E-26
FinnGen	rs6998881	8	23626444	C	T	0.336029	0.111834	0.0160183	2.92E-12
FinnGen	rs71379339	17	49187093	T	C	0.0146101	0.533753	0.0557909	1.10E-21
FinnGen	rs7222314	17	71108797	G	A	0.502431	-0.0949986	0.0151689	3.78E-10
FinnGen	rs7253360	19	38226624	G	A	0.552483	-0.0863985	0.0151958	1.30E-08
FinnGen	rs72725879	8	127091724	T	C	0.194846	0.108678	0.0188089	7.56E-09
FinnGen	rs72831686	17	47774344	A	G	0.0148398	0.455818	0.0565579	7.67E-16
FinnGen	rs73179053	22	43105614	C	T	0.0630229	-0.199062	0.032828	1.33E-09
FinnGen	rs75691080	20	63638397	T	C	0.113991	-0.145874	0.0247721	3.89E-09
FinnGen	rs7679673	4	105140377	A	C	0.488066	-0.0973147	0.0152562	1.79E-10
FinnGen	rs77541621	8	127064901	A	G	0.0245126	0.638415	0.0431078	1.27E-49
FinnGen	rs78059576	17	49217647	A	C	0.00415746	1.18851	0.0979014	6.50E-34
FinnGen	rs7819779	8	127196209	A	G	0.280742	-0.111397	0.0171383	8.04E-11
FinnGen	rs7820981	8	127387931	C	T	0.621207	0.105852	0.0158473	2.40E-11
FinnGen	rs78996488	8	127065335	T	C	0.0432688	-0.230453	0.0395273	5.54E-09
FinnGen	rs7940235	11	69052653	T	C	0.278436	0.0977194	0.0169521	8.19E-09
FinnGen	rs80181188	8	127125918	G	A	0.00630697	0.585227	0.0861452	1.09E-11
FinnGen	rs880411	17	37729291	C	T	0.148058	-0.14015	0.0219032	1.57E-10

FinnGen	rs9856752	3	87149239	G	A	0.529833	-0.0849992	0.0152099	2.29E-08

EA, effect allele; EAF, effect allele frequency; IL, interleukin; IL-1ra, IL-1 receptor antagonist; IL-2ra, IL-2 receptor alpha subunit; IL-6ra, IL-6 receptor subunit alpha; NEA, non-effect allele; Position, position based on hg_37; SNP, single nucleotide polymorphism.

Supplementary Table 4. F statistics and Power estimation

IL	Instruments	VE	F	PRACTICAL				UK Biobank				FinnGen			
				**N**	**Case%**	**OR ≤**	**OR ≥**	**N**	**Case%**	**OR ≤**	**OR ≥**	**N**	**Case%**	**OR ≤**	**OR ≥**
IL-1β	1	0.007	50	140,272	0.564	0.83	1.21	177,453	0.043	0.68	1.50	135,408	0.077	0.69	1.40
IL-1ra	4	0.096	221	140,272	0.564	0.95	1.05	177,453	0.043	0.91	1.11	135,408	0.077	0.91	1.10
IL-2ra	1	0.038	164	140,272	0.564	0.92	1.08	177,453	0.043	0.83	1.18	135,408	0.077	0.88	1.16
IL-6	2	0.026	118	140,272	0.564	0.91	1.10	177,453	0.043	0.79	1.23	135,408	0.077	0.82	1.20
IL-6ra	4	0.699	473	140,272	0.564	0.99	1.01	177,453	0.043	0.97	1.04	135,408	0.077	0.99	1.01
IL-8	2	0.010	47	140,272	0.564	0.85	1.15	177,453	0.043	0.76	1.35	135,408	0.077	0.78	1.34
IL-16	8	0.289	529	140,272	0.564	0.97	1.03	177,453	0.043	0.96	1.06	135,408	0.077	0.97	1.06
IL-17	1	0.010	40	140,272	0.564	0.85	1.15	177,453	0.043	0.76	1.35	135,408	0.077	0.78	1.34
IL-18	8	0.121	138	140,272	0.564	0.96	1.04	177,453	0.043	0.91	1.10	135,408	0.077	0.91	1.09
IL-27	11	0.430	485	140,272	0.564	0.97	1.03	177,453	0.043	0.96	1.05	135,408	0.077	0.95	1.05

GWAS, genome-wide association studies; IL, interleukin; IL-1ra, IL-1 receptor antagonist; IL-2ra, IL-2 receptor alpha subunit; IL-6ra, IL-6 receptor subunit alpha; VE, variance explained; OR, odds ratio. ORs were estimated by setting 80% of power.

Supplementary Table 5. Associations of genetically predicted interleukins' levels with prostate cancer in sensitivity analyses

	Used SNPs	Practical			UK Biobank			FinnGen		
		OR	95% CI	P	OR	95% CI	P	OR	95% CI	P
**IL-1β**										
Weighted median	1	-	-	-	-	-	-	-	-	-
MR-Egger	1	-	-	-	-	-	-	-	-	-
MR-RAPS	1	1.36	1.10-1.67	0.004	0.96	0.62-1.48	0.851	0.87	0.51-1.48	0.614
**IL-1ra**										
Weighted median	4	0.91	0.86-0.97	0.002	1.04	0.90-1.19	0.566	0.98	0.87-1.10	0.696
MR-Egger	4	0.95	0.83-1.10	0.577	1.54	1.14-2.07	0.105	1.01	0.77-1.32	0.941
MR-RAPS	4	0.91	0.86-0.96	0.001	1.04	0.87-1.24	0.657	0.96	0.86-1.07	0.470
**IL-2ra**										
Weighted median	1	-	-	-	-	-	-	-	-	-
MR-Egger	1	-	-	-	-	-	-	-	-	-
MR-RAPS	1	0.99	0.95-1.03	0.615	1.02	0.92-1.12	0.738	1.00	0.90-1.10	0.934
**IL-6**										
Weighted median	2	-	-	-	-	-	-	-	-	-
MR-Egger	2	-	-	-	-	-	-	-	-	-
MR-RAPS	2	1.10	1.00-1.20	0.045	1.03	1.00-1.05	0.035	1.05	0.88-1.26	0.562
**IL6-ra**										
Weighted median	4	1.02	1.00-1.03	0.037	1.03	0.99-1.06	0.109	1.01	0.98-1.04	0.632
MR-Egger	4	1.02	0.98-1.07	0.439	1.03	0.97-1.08	0.444	1.03	0.92-1.15	0.640
MR-RAPS	4	1.02	1.00-1.03	0.044	1.03	0.99-1.06	0.127	1.01	0.97-1.04	0.713
**IL-8**										
Weighted median	2	-	-	-	-	-	-	-	-	-
MR-Egger	2	-	-	-	-	-	-	-	-	-
MR-RAPS	2	0.92	0.68-1.26	0.620	0.99	0.73-1.36	0.974	0.93	0.68-1.28	0.662
**IL-16**										
Weighted median	6	0.99	0.96-1.03	0.809	1.00	0.94-1.06	0.906	0.98	0.92-1.05	0.645
MR-Egger	6	0.99	0.95-1.04	0.887	1.01	0.91-1.11	0.920	0.97	0.90-1.06	0.582
MR-RAPS	6	1.00	0.95-1.04	0.888	0.97	0.80-1.17	0.748	0.99	0.92-1.06	0.683
**IL-17**										
Weighted median	1	-	-	-	-	-	-	-	-	-
MR-Egger	1	-	-	-	-	-	-	-	-	-
MR-RAPS	1	1.06	0.89-1.26	0.507	1.05	0.75-1.47	0.792	0.90	0.66-1.23	0.501
**IL-18**										

Weighted median	7	1.00	0.94-1.06	0.938	0.99	0.87-1.12	0.844	0.98	0.87-1.11	0.734
MR-Egger	8	0.92	0.81-1.06	0.291	0.99	0.78-1.25	0.923	1.12	0.89-1.40	0.383
MR-RAPS	8	0.99	0.93-1.07	0.868	1.02	0.92-1.12	0.765	0.97	0.87-1.09	0.634
**IL-27**										
Weighted median	11	0.99	0.96-1.01	0.308	1.00	0.95-1.05	0.892	0.98	0.94-1.03	0.436
MR-Egger	11	1.00	0.95-4.04	0.878	1.01	0.94-1.08	0.865	0.99	0.92-1.06	0.729
MR-RAPS	11	0.98	0.95-1.02	0.381	0.99	0.94-1.04	0.748	0.99	0.95-1.04	0.651

IL, interleukin; IL-1ra, IL-1 receptor antagonist; IL-6ra, IL-6 receptor subunit alpha; OR, odds ratio; CI, confidence interval; SNPs, single nucleotide polymorphisms.

Supplementary Table 6. I^2_{GX}, MR-Egger intercept, Simulation extrapolation (SIMEX) for genetically predicted interleukins' levels with prostate cancer

	Used SNPs	Cochrane's Q	I^2	I^2_{GX}	MR-Egger_intercept			Simulation extrapolation (SIMEX)		
					Intercept	SE	P	Intercept	SE	P
**IL-1ra**										
PRACTICAL	4	0.609	0.0	0.96	-0.008	0.012	0.559	-	-	-
UK Biobank	4	0.027	67.2	0.97	-0.071	0.025	0.102	-	-	-
FinnGen	4	0.585	0	0.97	-0.008	0.022	0.728	-	-	-
**IL-6**										
PRACTICAL	2	0.913	0.0	0.96	-	-	-	-	-	-
UK Biobank	2	0.929	0.0	0.97	-	-	-	-	-	-
FinnGen	2	0.442	0.0	0.97	-	-	-	-	-	-
**IL-6ra**										
PRACTICAL	4	0.166	40.8	1	-0.003	0.013	0.850	-	-	-
UK Biobank	4	0.626	0.0	0.99	0.001	0.017	0.962	-	-	-
FinnGen	4	0.012	72.6	0.99	-0.019	0.033	0.613	-	-	-
**IL-8**										
PRACTICAL	2	0.003	88.0	1	-	-	-	-	-	-
UK Biobank	2	0.385	0.0	1	-	-	-	-	-	-
FinnGen	2	0.678	0.0	0.99	-	-	-	-	-	-
**IL-16**										
PRACTICAL	6	0.411	0.6	0.99	-0.002	0.006	0.802	-	-	-
UK Biobank	6	0.129	39.4	1	-0.006	0.015	0.690	-	-	-
FinnGen	6	0.683	0	0.99	0.005	0.012	0.669	-	-	-
**IL-18**										
PRACTICAL	7	0.052	49.7	0.97	0.016	0.010	0.270	-	-	-
UK Biobank	8	0.300	16.5	0.97	0.005	0.018	0.776	-	-	-
FinnGen	8	0.433	0	0.97	-0.023	0.016	0.237	-	-	-
**IL-27**										
PRACTICAL	11	0.017	53.5	0.99	-0.003	0.006	0.556	-	-	-
UK Biobank	11	0.563	0.0	0.99	-0.006	0.008	0.491	-	-	-
FinnGen	11	0.356	9.4	0.99	0.001	0.009	0.986	-	-	-

IL, interleukin; IL-1ra, IL-1 receptor antagonist; IL-6ra, IL-6 receptor subunit alpha; OR, odds ratio; SE, standard error; SNPs, single nucleotide polymorphisms.

Supplementary Table 7. The results of MR-PRESSO

Exposure	MR-PRESSO (Raw)			MR-PRESSO (Outlier-corrected)			*P* for global test	*P* for distortion test[b]
	BETA	SE	*P*	BETA	SE	*P*		
**IL-1ra**								
PRACTICAL	-0.091	0.058	0.523	-	-	-	0.051	-
UK Biobank	0.0255	0.099	0.813	-	-	-	0.131	-
FinnGen	-0.0392	0.042	0.423	-	-	-	0.234	-
**IL-6ra**								
PRACTICAL	0.017	0.010	0.215	-	-	-	0.594	-
UK Biobank	0.0275	0.013	0.126	-	-	-	0.631	-
FinnGen	0.00455	0.031	0.893	-	-	-	0.376	-
**IL-16**								
PRACTICAL	-0.006	0.016	0.689	-	-	-	0.564	-
UK Biobank	-0.007	0.037	0.854	-	-	-	0.512	-
FinnGen	-0.014	0.026	0.609	-	-	-	0.854	-
**IL-18**								
PRACTICAL	-0.005	0.033	0.874	-	-	-	0.076	-
UK Biobank	0.019	0.052	0.715	-	-	-	0.340	-
FinnGen	-0.031	0.049	0.561	-	-	-	0.532	-
**IL-27**								
PRACTICAL	-0.013	0.017	0.441	-	-	-	0.203	-
UK Biobank	-0.011	0.024	0.659	-	-	-	0.680	-
FinnGen	-0.012	0.024	0.627	-	-	-	0.537	-

SE, standard error;
No outliers were detected in MR-PRESSO. Thus, MR-PRESSO derived estimates were identical to that of the inverse variance weighted analyses.

Supplementary Table 8. Traits associated with ILs-associated SNPs in PhenoScanner V2 at the genome-wide significance level ($P<5\times10^{-8}$)

IL	SNP	Gene	EA	Associated traits	Direction	P value
IL-1β	rs6917603	*HLA locus*	T	Lipid metabolism	NA	3.00E-29
IL-1ra	rs6734238	*IL1F10*	A	Sum neutrophil eosinophil counts	-0.034	3.50E-21
				Granulocyte count	-0.034	6.74E-21
				Fibrinogen levels	0.009	6.00E-19
				White blood cell count	-0.031	1.00E-17
				Myeloid white cell count	-0.034	4.19E-20
				Lymphocyte percentage of white cells	0.021	5.82E-09
				Neutrophil count	-0.034	1.13E-20
				Sum basophil neutrophil counts	-0.034	1.64E-20
				C reactive protein levels	-0.050	2.00E-17
				Neutrophil percentage of white cells	-0.023	2.87E-10
IL-1ra	rs4848312	*IL36B*	A	NA	NA	NA
IL-1ra	rs149418433	*PSMD3*	A	NA	NA	NA
IL-1ra	rs62143194	*NLRP12*	C	Monocyte percentage of white cells	0.081	2.64E-74
				Monocyte count	0.072	3.74E-58
				Granulocyte percentage of myeloid white cells	-0.070	1.05E-55
IL-2ra	rs12722497	*IL2RA*	A	NA	NA	NA
IL-6	rs2228145	*IL6R*	A	Cerebrospinal fluid biomarker levels	NA	7.00E-29
				Hayfever, allergic rhinitis or eczema	-0.008	3.69E-14
				Coronary artery disease	0.039	4.80E-14
				Fibrinogen	-5.300	2.00E-11
IL-6	rs4959106	*HLA-DQA1*	T	Self-reported malabsorption or coeliac disease	0.004	1.58E-168
				Treatment with methotrexate	-0.002	2.08E-41
				White blood cell count	-0.044	5.56E-40
				Sum eosinophil basophil counts	-0.040	3.19E-32
				Eosinophil count	-0.039	3.29E-32
				Myeloid white cell count	-0.037	9.22E-28
				Sum neutrophil eosinophil counts	-0.035	2.62E-25
				Sitting height	0.019	5.84E-23

				Granulocyte count	-0.035	1.11E-25
				Lymphocyte count	-0.031	9.67E-21
				Treatment with sulfasalazine	-0.001	2.21E-20
				Sum basophil neutrophil counts	-0.033	2.92E-22
				Neutrophil count	-0.032	8.14E-22
				Self-reported multiple sclerosis	0.001	1.21E-21
				Trunk fat-free mass	0.012	1.96E-15
				Treatment with folic acid product	-0.002	3.35E-15
				Self-reported ankylosing spondylitis	-0.001	7.47E-19
				Treatment with insulin product	-0.002	1.89E-17
				Monocyte count	-0.027	1.52E-15
				Trunk predicted mass	0.012	1.90E-15
				Reticulocyte count	-0.023	4.04E-12
				Basal metabolic rate	0.011	1.58E-11
				Hand grip strength right	0.014	1.26E-14
				Self-reported sarcoidosis	0.001	2.25E-14
				Hayfever, allergic rhinitis or eczema	0.007	2.22E-12
				Self-reported type 1 diabetes	0.000	1.57E-09
				Self-reported high cholesterol	-0.004	4.90E-08
				Forced vital capacity	0.013	1.50E-10
				IgA deficiency	0.279	4.99E-10
IL-6ra	rs12126142	*IL6R*	A	Hayfever, allergic rhinitis or eczema	0.008	7.27E-14
				C-reactive protein	-0.100	2.51E-10
				Allergic disease	0.037	1.97E-10
				Coronary artery disease	-0.037	1.23E-10
				Monocyte percentage of white cells	0.022	9.53E-10
IL-6ra	rs36106412	*NUP210L*	A	NA	NA	NA
IL-6ra	rs4845661	*KCNN3*	C	NA	NA	NA
IL-6ra	rs7258728	*CYP2A7*	C	NA	NA	NA
IL-8	rs7655660	*CXCL8*	A	NA	NA	NA

IL-8	rs972492	*GDNF-AS1*	A	NA	NA	NA
IL-16	rs58623354	*GRK7*	T	NA	NA	NA
IL-16	rs774284803	*ADAMTSL3*	A	NA	NA	NA
IL-16	rs4778639	*IL16*	T	NA	NA	NA
IL-16	rs859	*IL16*	A	NA	NA	NA
IL-16	rs1564641	*STARD5*	A	NA	NA	NA
IL-16	rs117089718	*MLLT3*	T	NA	NA	NA
IL-16	rs150568717	*AGBL1*	T	NA	NA	NA
IL-16	rs10424405	*NLRP12*	A	Monocyte percentage of white cells	0.081	8.83E-76
				Monocyte count	0.072	1.44E-59
				Granulocyte percentage of myeloid white cells	-0.071	9.46E-58
IL-17	rs1530455	*PDIA5*	T	Platelet distribution width	-0.043	5.05E-31
				Platelet count	0.039	7.39E-25
				Plateletcrit	0.023	2.22E-09
				Mean platelet volume	-0.042	3.11E-29
IL-18	rs385076	*NLRC4*	T	NA	NA	NA
IL-18	rs9867857	*LEKR1*	T	Heel bone mineral density right	0.027	7.98E-11
				Heel bone mineral density	0.019	1.93E-09
				Heel bone mineral density left	0.027	1.48E-10
IL-18	rs17229943	*RAD17*	A	NA	NA	NA
IL-18	rs916964	*NFE2L3*	C	NA	NA	NA
IL-18	rs4606077	*NAPRT*	T	NA	NA	NA
IL-18	rs5002812	*CARD18*	A	NA	NA	NA
IL-18	rs5744249	*IL18*	A	Heel bone mineral density	-0.020	2.68E-08
IL-18	rs10424405	*NLRP12*	A	Monocyte percentage of white cells	0.081	8.83E-76
				Monocyte count	0.072	1.44E-59
				Granulocyte percentage of myeloid white cells	-0.071	9.46E-58
IL-27	rs1257169	*MGAT5*	A	NA	NA	NA
IL-27	rs9715769	*TLR10*	A	Hayfever, allergic rhinitis or eczema	0.021	4.46E-51
				Asthma	0.007	1.99E-11
				Lymphocyte count	0.033	3.06E-11
				Allergic disease	0.091	6.12E-32

IL-27	rs11711157	*CPN2*	T	NA	NA	NA
IL-27	rs11599750	*CPN1*	T	Height	-0.023	2.90E-14
IL-27	rs10774624	*SH2B3*	A	Plateletcrit	-0.109	8.23E-193
				Eosinophil count	-0.100	2.93E-168
				Platelet count	-0.100	1.85E-161
				Basal metabolic rate	0.014	5.23E-18
				Hip circumference	0.016	9.49E-11
				Past tobacco smoking	0.019	2.00E-09
				Lymphocyte count	-0.088	1.38E-130
				Reticulocyte count	-0.072	1.07E-87
				Self-reported hypertension	-0.011	2.86E-23
				Vitiligo	-0.239	6.00E-23
				White blood cell count	-0.062	1.61E-66
				Hematocrit	-0.060	2.61E-63
				Birth weight	0.029	4.75E-19
				Self-reported hypothyroidism or myxoedema	-0.009	1.35E-70
				Hemoglobin concentration	-0.062	5.47E-68
				Trunk predicted mass	0.015	1.76E-21
				Trunk fat-free mass	0.015	2.38E-21
				Diastolic blood pressure	-0.037	1.27E-49
				Coronary artery disease	-0.064	1.73E-26
IL-27	rs966541	*ERGIC2*	A	Height	-0.017	1.92E-18
				Comparative height size at age 10	-0.013	1.38E-12
IL-27	rs111072793	*ASGR1*	A	NA	NA	NA
IL-27	rs704	*VTN*	A	Osteoprotegerin levels	0.030	1.00E-09
IL-27	rs4905	*EBI3*	A	NA	NA	NA
IL-27	rs59975010	*ZNF799*	T	NA	NA	NA
IL-27	rs12625762	*ERGIC3*	A	Trunk fat-free mass	0.024	1.87E-26
				Height	0.030	3.69E-33
				Basal metabolic rate	0.023	5.42E-22

EA, effect allele; IL, interleukin; IL-1ra, IL-1 receptor antagonist; IL-2ra, IL-2 receptor alpha subunit; IL-6ra, IL-6 receptor subunit alpha; SNP, single nucleotide polymorphism.

Supplementary Table 9. Associations of genetically predicted interleukins' levels with prostate cancer after exception for body mass, smoking and high cholesterol

	Used SNPs	PRACTICAL			UK Biobank			FinnGen		
		OR	95% CI	P	OR	95% CI	P	OR	95% CI	P
**IL-6**										
Weighted median	1	-	-	-	-	-	-	-	-	-
MR-Egger	1	-	-	-	-	-	-	-	-	-
MR-RAPS	1	1.09	0.99-1.21	0.079	1.16	0.94-1.42	0.164	1.02	0.84-1.24	0.864
**IL-27**										
Weighted median	10	0.99	0.96-1.01	0.320	1.00	0.95-1.05	0.884	0.98	0.94-1.03	0.443
MR-Egger	10	0.99	0.95-1.04	0.697	1.01	0.95-1.09	0.709	0.98	0.92-1.04	0.454
MR-RAPS	10	0.99	0.95-1.02	0.435	0.99	0.94-1.04	0.673	0.99	0.95-1.04	0.761

IL, interleukin; IL-1ra, IL-1 receptor antagonist; IL-6ra, IL-6 receptor subunit alpha; OR, odds ratio; CI, confidence interval; SNPs, single nucleotide polymorphisms.

Supplementary Table 10. Associations of genetically predicted prostate cancer with interleukins' levels in sensitivity analyses

	Used SNPs	Weighted median			MR-Egger			MR-RAPS		
		**BETA**	**95% CI**	**P**	**BETA**	**95% CI**	**P**	**BETA**	**95% CI**	**P**
**IL-1ra**										
Practical	109	-0.028	-0.038,0.014	0.361	0.009	-0.035,0.044	0.834	-0.025	-0.029,0.007	0.221
UK Biobank	31	-0.017	-0.029,0.014	0.501	-0.069	-0.078,0.018	0.232	-0.008	-0.019,0.013	0.691
FinnGen	88	-0.005	-0.012,0.017	0.750	-0.004	-0.015,0.011	0.777	-0.004	-0.010,0.007	0.716
**IL-6**										
Practical	109	-0.031	-0.046,0.019	0.430	-0.031	-0.061,0.035	0.587	-0.009	-0.025,0.017	0.729
UK Biobank	31	0.025	-0.015,0.037	0.395	-0.055	-0.084,0.037	0.443	0.022	-0.009,0.029	0.316
FinnGen	88	0.017	-0.009,0.025	0.383	-0.003	-0.017,0.014	0.854	-0.006	-0.013,0.008	0.614

IL, interleukin; IL-1ra, IL-1 receptor antagonist; CI, confidence interval; SNPs, single nucleotide polymorphisms.

Supplementary Table 11. I^2_{GX}, MR-Egger intercept, Simulation extrapolation (SIMEX) for genetically predicted prostate cancer with interleukins' levels

	Used SNPs	Cochrane's Q	I^2	I^2_{GX}	MR-Egger_intercept			Simulation extrapolation (SIMEX)		
					**Intercept**	**SE**	**P**	**Intercept**	**SE**	**P**
**IL-1ra**										
PRACTICAL	109	0.840	0.0	0.68	-0.003	0.003	0.422	0.044	0.025	0.084
UK Biobank	31	0.575	0.0	0.52	0.008	0.007	0.264	-0.049	0.026	0.061
FinnGen	88	0.711	0.0	0.86	0.001	0.003	0.939	-0.009	0.015	0.545
**IL-6**										
PRACTICAL	109	0.780	0.0	0.94	0.002	0.004	0.637			
UK Biobank	31	0.300	10.9	0.69	0.001	0.010	0.356	0.004	0.041	0.925
FinnGen	88	0.773	0	0.88	-0.001	0.003	0.968	-0.008	0.019	0.642

IL, interleukin; IL-1ra, IL-1 receptor antagonist; IL-6ra, IL-6 receptor subunit alpha; SE, standard error; SNPs, single nucleotide polymorphisms.
